# Unveiling the impact of interprofessional education on shaping students’ interprofessional identity and collaboration perception: a mixed-method study

**DOI:** 10.1186/s12909-024-05833-0

**Published:** 2024-08-08

**Authors:** Qing He, John Ian Wilzon T. Dizon, Fraide A. Ganotice, Binbin Zheng, Pauline Pui Ning Yeung, Xiaoai Shen, Lily Yuen Wah Ho, Arkers Kwan Ching Wong, Franco Wing Tak Cheng, Karen Man Kei Chan, Linda Chan, Sarah So Ching Chan, Amy Yin Man Chow, Jody Kwok Pui Chu, Denise Mae Chua, Edwin Chung-Hin Dung, Wei-Ning Lee, Feona Chung Yin Leung, Qun Wang, Kevin K. Tsia, Dana Vackova, Julienne Jen, George L. Tipoe

**Affiliations:** 1 Bau Institute of Medical and Health Sciences Education, Li Ka Shing Faculty of Medicine, The University of Hong Kong, Hong Kong SAR, China; 2 School of Clinical Medicine, Li Ka Shing Faculty of Medicine, The University of Hong Kong, Hong Kong SAR, China; 3 School of Nursing, Faculty of Health and Social Sciences, The Hong Kong Polytechnic University, Hong Kong SAR, China; 4 Department of Pharmacology and Pharmacy, Li Ka Shing Faculty of Medicine, The University of Hong Kong, Hong Kong SAR, China; 5 Swallowing Research Laboratory, Faculty of Education, The University of Hong Kong, Hong Kong SAR, China; 6 Department of Social Work and Social Administration, Faculty of Social Sciences, The University of Hong Kong, Hong Kong SAR, China; 7 Department of Electrical and Electronic Engineering, Faculty of Engineering, The University of Hong Kong, Hong Kong SAR, China; 8 School of Chinese Medicine, Li Ka Shing Faculty of Medicine, The University of Hong Kong, Hong Kong SAR, China; 9 Department of Mechanical Engineering, Faculty of Engineering, The University of Hong Kong, Hong Kong SAR, China; 10 School of Public Health, Li Ka Shing Faculty of Medicine, The University of Hong Kong, Hong Kong SAR, China; 11 Department of Professional Legal Education, Faculty of Law, The University of Hong Kong, Hong Kong SAR, China

**Keywords:** Interprofessional education, Interprofessional identity, Collaboration perception, Health professions education, Medical education

## Abstract

**Background:**

Interprofessional education (IPE) has the potential to shape students’ collaboration perception and interprofessional identity but remains understudied. This study aims to understand the effects of the IPE program as a contextual trigger to promote collaboration perception change and interprofessional identity formation among healthcare professional students.

**Methods:**

Using concurrent triangulation mixed-methods, we examined the relationship between collaboration perception and interprofessional identity change among health profession students (*N* = 263), and explored their perspectives on how their IPE experiences influenced their perception and identity. Participants completed the Interdisciplinary Education Perception Scale and Extended Professional Identity Scale and responded to open-ended questions before and after the IPE intervention. Pearson’s correlation, t-tests, regression (quantitative), and thematic analysis (qualitative) were conducted.

**Results:**

Teams with initially lower collaboration perception (M = 3.59) and lower interprofessional identity (M = 3.59) showed a significant increase in collaboration perception (M = 3.76, t = 2.63; *p* = .02) and interprofessional identity (M = 3.97, t = 4.86; *p* < .001) after participating in IPE. The positive relationship between collaboration perception and interprofessional identity strengthened after participating in IPE, as evident from the correlation (Time 1: *r* = .69; *p* < .001; Time 2: *r* = .79; *p* < .001). Furthermore, collaboration perception in Time 1 significantly predicted the variance in interprofessional identity at Time 2 (β = 0.347, *p* < .001). Qualitative findings indicated that 85.2% of students expressed that IPE played a role in promoting their interprofessional identity and collaboration attitudes.

**Conclusions:**

Incorporating the IPE program into the curriculum can effectively enhance students’ collaboration perception and interprofessional identity, ultimately preparing them for collaborative practice in the healthcare system. By engaging students in interprofessional teamwork, communication, and joint decision-making processes, the IPE program provides a valuable context for students to develop a sense of belonging and commitment to interprofessional collaboration.

**Supplementary Information:**

The online version contains supplementary material available at 10.1186/s12909-024-05833-0.

## Background

Professional identity is a critical aspect of professional practice, influencing how individuals perceive, present, and conduct themselves within their respective professions [1–3]. Extensive research in medical education has explored the formation of professional identity through the internalization of behavior, norms, values, and standards within specific professional communities [1–8]. However, there is also a growing interest in interprofessional identity within the medical education field, [7, 9–12] which is defined as “the development of a robust cognitive, psychological, and emotional sense of belonging to an interprofessional community, necessary to achieve shared context-dependent goals” [7](p6). This emerging interest in interprofessional identity is driven by the recognition of the advantages of interprofessional team-based patient management. Despite this increasing interest, the topic of interprofessional identity remains understudied and requires further investigation.

Interprofessional education (IPE) has become an integral component in many health profession education programs, fostering the development of norms, values, and standards that contribute to professional identity [13]. Recent research found a positive effect of interprofessional identity on interprofessional collaboration following an IPE course [10]. The extended professional identity theory (EPIT) [14], drawing from identity theory and social identity theory [15], proposes that interprofessional identity functions as a broader social identity associated with belonging to a larger group, triggered by specific contextual factors. Individuals can identify triggers that activate the professional identities of other disciplines, forming a higher-level trigger that activates their interprofessional identity [10]. The EPIT [14] proposes three dimensions of interprofessional identity, namely interprofessional belonging, interprofessional commitment, and interprofessional beliefs, and highlights the role of socialization in shaping the collective dimension of an individual’s identity. By shifting the focus from professional to interprofessional identity, the process of interprofessional socialization can promote the formation of interprofessional identity and facilitate collaboration attitudes and behaviors among students [14]. Given the significance of interprofessional identity in IPE and collaborative practice [16], some scholars [7, 17] advocate for tracking its development over time using a mixed-method approach to bridge the gap between literature and practice.

As identity formation is influenced by individual perceptions [18], previous studies have investigated collaboration perception and interprofessional identity concurrently [7, 9]. However, the conceptual link between these two constructs requires thorough examination in the IPE setting, and the specific role of IPE as an activating contextual trigger for promoting collaboration perception and interprofessional identity remains understudied. Therefore, exploring how an IPE intervention can promote collaboration perception and interprofessional identity among health profession students while exploring the relationship between these constructs, can contribute to the research literature.

In response, the present study aims to investigate the impact of an IPE PRAE intervention (Preparation → Readiness Assurance → Application Exercise → Enrichment Activity) on changes in collaboration perception and interprofessional identity among health profession students. To achieve these aims, a mixed-method approach was utilized. This study was motivated by the need to clarify interprofessional identity formation, the interplay between interprofessional identity and professional identity/competencies, and the contextual factors in the learning and work environment [10]. Therefore, we hypothesized that there would be a significant improvement in students’ collaboration perception and interprofessional identity after the IPE intervention (H_1_), and there would be a positive relationship between students’ collaboration perception and interprofessional identity (H_2_).

## Methods

### Context

**Fig. 1:**
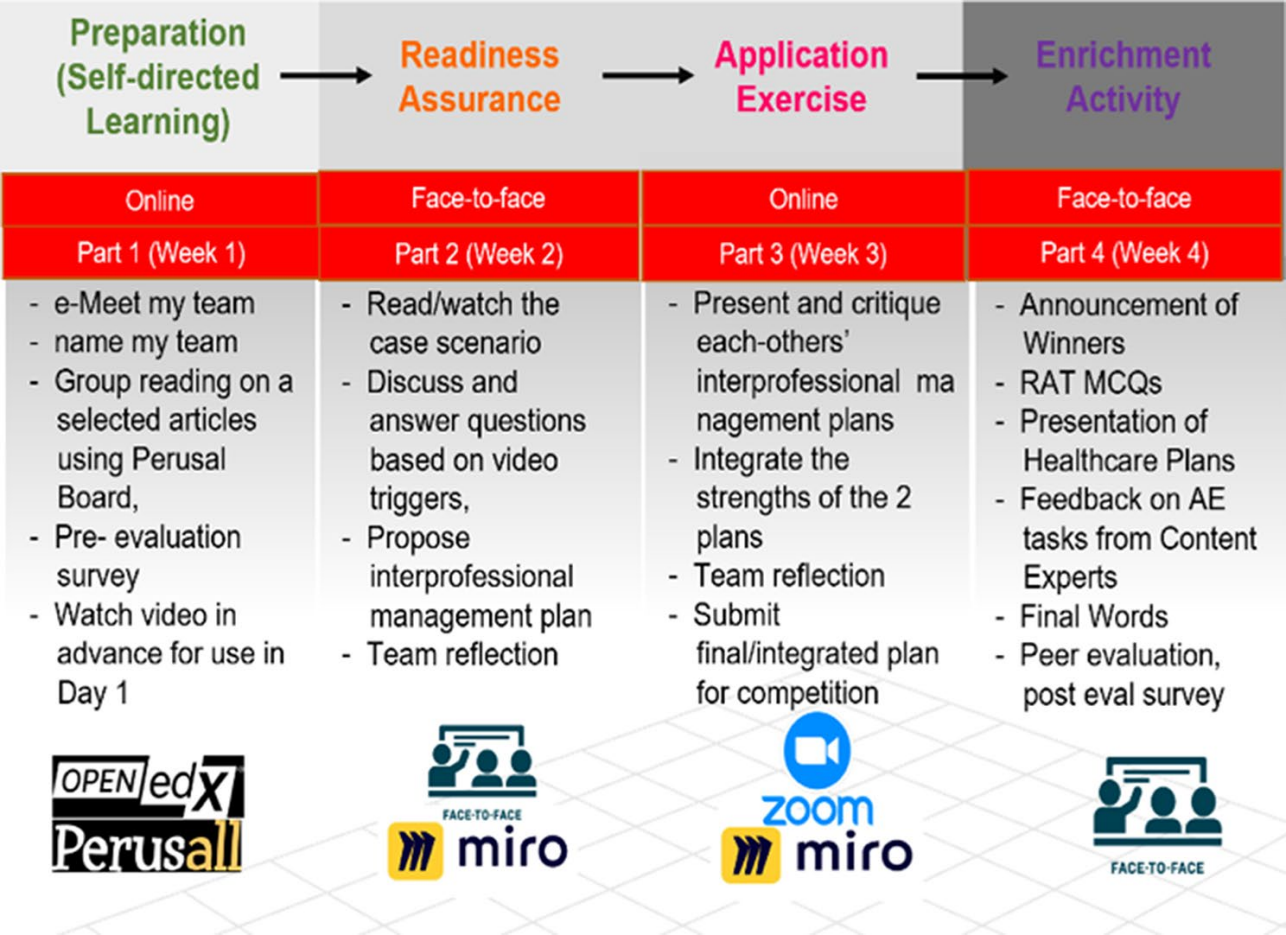
The IPE PRAE Implementation Model. *Note* IPE: Interprofessional Education; PRAE: Preparation, Readiness assurance, Application exercise, Enrichment activity; RAT MCQs: Readiness Assurance Test Multiple Choice Questions; AE: Application Exercise

This study originated from an IPE project conducted at a university in Hong Kong. The primary goal of the IPE project was to foster collaboration among students pursuing healthcare professions. The specific teaching module focused on the management of emerging infection control cases, and throughout the teaching module, learners were organized into various IPE teams. Each team consisted of approximately 10 individuals representing different disciplines. This IPE PRAE aligns with Mitchell et al.’s [19] recommendation to develop shared goals, vision, and interdependence within interprofessional teams (Fig. 1).

### Participants and procedures

We adopted a concurrent triangulation mixed-methods approach to confirm, cross-validate, and corroborate findings by integrating both quantitative and qualitative studies at the same time rather than having one inform the collection of the other [20]. Combining quantitative and qualitative methods allows the findings to be elucidated in comprehensive way [21].

In September 2022, a cohort of students enrolled in the IPE course was invited to take part in an online survey. The survey links were provided to individual students via mass email at the beginning of the IPE program implementation week (pre-test) and after the program (post-test), which included both quantitative and qualitative survey items. Prior to their voluntary participation in the study, informed consent was obtained from all participants. The study received ethical approval from the Institutional Human Research Ethics Committee (EA210433), ensuring compliance with ethical guidelines.

Among the 357 students who were invited to participate in this study, a total of 263 undergraduate health profession students from two universities in Hong Kong participated (73.7% response rate). The majority of the participants were females (64%) and the mean age was 21.4 years (*SD* = 1.81) with an age range of 18–28 years. Most of the participants were in their Year 5 (39.5%) while others were from Year 4 (21.3%), Year 3 (32.3%), and Year 2 (6.8%), respectively (Table 1). Participants were from Chinese Medicine (*n* = 8), Biomedical Engineering (*n* = 18), Law (*n* = 17), Medicine (*n* = 95), Nursing (*n* = 72), Social Work (*n* = 31), and Speech and Hearing Sciences (*n* = 22).

**Table 1:** Participants characteristics (*n* = 263)

Gender	*n*	%
Female	168	63.88
Male	95	36.12
**Discipline**		
Chinese Medicine	8	3.04
Biomedical Engineering	18	6.84
Law	17	6.46
Medicine	95	36.12
Nursing	72	27.38
Social Work	31	11.79
Speech and Hearing Sciences	22	8.37
**Year Level**		
Year II	18	6.84
Year III	85	32.32
Year IV	56	21.29
Year V	104	39.54

*Note* Mean age of the participants was 21.4 years (*SD* = 1.81)

### Measures

#### Interdisciplinary collaboration perception

We used the 18-item Interdisciplinary Education Perception Scale (IEPS) [22] to measure the collaboration perceptions of participants who are exposed to interdisciplinary settin*gs.* Sample items include, “*Individuals in my profession need to cooperate with other professions*” to which participants can rate their agreement to such statements using the 5-point Likert scale that ranges from “strongly disagree” (1) to “strongly agree” (5). In this study, we used the whole scale to generate a global interdisciplinary perception score (pre-test α = 0.96; post-test α = 0.97). Higher mean scores on the scale indicate a greater perception of interdisciplinary collaboration.

#### Interprofessional identity

The 12-item Extended Professional Identity Scale (EPIS) [14] was used to measure interprofessional identity. The EPIS has three dimensions: interprofessional belonging, e.g., “*I like meeting and getting to know people from other health professions*”, (pre-test α = 0.91; post-test α = 0.94), interprofessional commitment, e.g., “*I would be very happy to spend the rest of my career with an interprofessional team*” (pre-test α = 0.90; post-test α = 0.96), and interprofessional beliefs, e.g., “*Interprofessional team members should jointly agree to communicate plans for patient care*” (pre-test α = 0.93; post-test α = 0.95). Participants responded to the items using a five-point Likert scale ranging from “1 = strongly disagree” to “5 = strongly agree”. Higher mean scores on the overall scale imply greater interprofessional identity and higher mean scores on the individual subscales indicate greater propensity for aligning oneself to any of the three subscales.

#### Open-ended questions

We used one open-ended question in the pre-test and two questions in the post-test to supplement the quantitative measures we indicated above and evaluated the feasibility and acceptability of the program. In the pre-test, we asked the participants the question, “*What is the most important thing you hope to learn in IPE?*”. In the post-test, we asked the participants the following questions: (1) “*Did the whole learning process meet your expectations? If not*,* could you describe some reasons?*”, (2) “*What was the most beneficial aspect of IPE?*”

### Data analysis

Quantitative analysis. Paired t-tests were used to test for the pre-and post-test mean difference. To test the relationship between pre- and post-test interdisciplinary collaboration perception and interprofessional identity, we used pairwise Pearson’s correlation and linear regression. Given that students were nested within teams, we also explored the students’ team-level collaboration perception and interprofessional identity changes. Following the procedure in previous studies [23], we calculated each team’s mean global collaboration perception and interprofessional identity in the pre-test and ranked them from highest (1) to lowest (40) where higher-performing team ranking indicates greater interprofessional identity and collaboration perception. Subsequently, we employed an independent t-test to examine the differences between higher-performing teams (Teams ranked 1 to 20) and lower-performing teams (Teams ranked 21 to 40) on interdisciplinary perception and interprofessional identity separately in Time 1 and Time 2. Analyses were performed using the Statistical Package for Social Sciences (SPSS, Version 28) [24].

Qualitative analysis. Following an interpretive approach, two researchers conducted a thematic analysis by using NVIVO software program version 12 to analyze the qualitative findings, aiming to achieve the credibility, rigor, and trustworthiness of the qualitative findings [25]. In the initial phase, two researchers independently performed inductive coding on a selection of open-ended responses and identified preliminary themes. This involved closely reading and familiarizing themselves with the data, generating initial codes, and grouping them to form potential themes. The researchers engaged in regular discussions to compare and refine their coding decisions, establishing consensus on the initial code system. In the subsequent phase, the researchers systematically applied the initial code system to the remaining open-ended responses, continuously reviewing and revising the codes and themes as new insights emerged. This iterative process allowed for the identification of additional themes and sub-themes. The researchers collaborated closely to ensure consistency in the identified themes, sub-themes, and codes, discussing any discrepancies and reaching consensus through thorough deliberation. In the final stage of analysis, consensus was reached between the two researchers by refining the themes and codes based on their comprehensive understanding of the data. The refined themes and codes were then applied in the third round of coding for the text responses, ensuring a consistent and comprehensive analysis. Furthermore, the research team upheld reflexivity in their analysis by engaging in discussions regarding the established assumptions. This approach was implemented to mitigate bias and guarantee the study’s credibility and rigor [26]. To present the qualitative research findings, the study adhered to the Standards for Reporting Qualitative Research, showcasing a dedicated effort to upholding the study’s integrity and trustworthiness.

## Results

### Changes in student-level collaboration perception and interprofessional identity before and after IPE PRAE

**Fig. 2:**
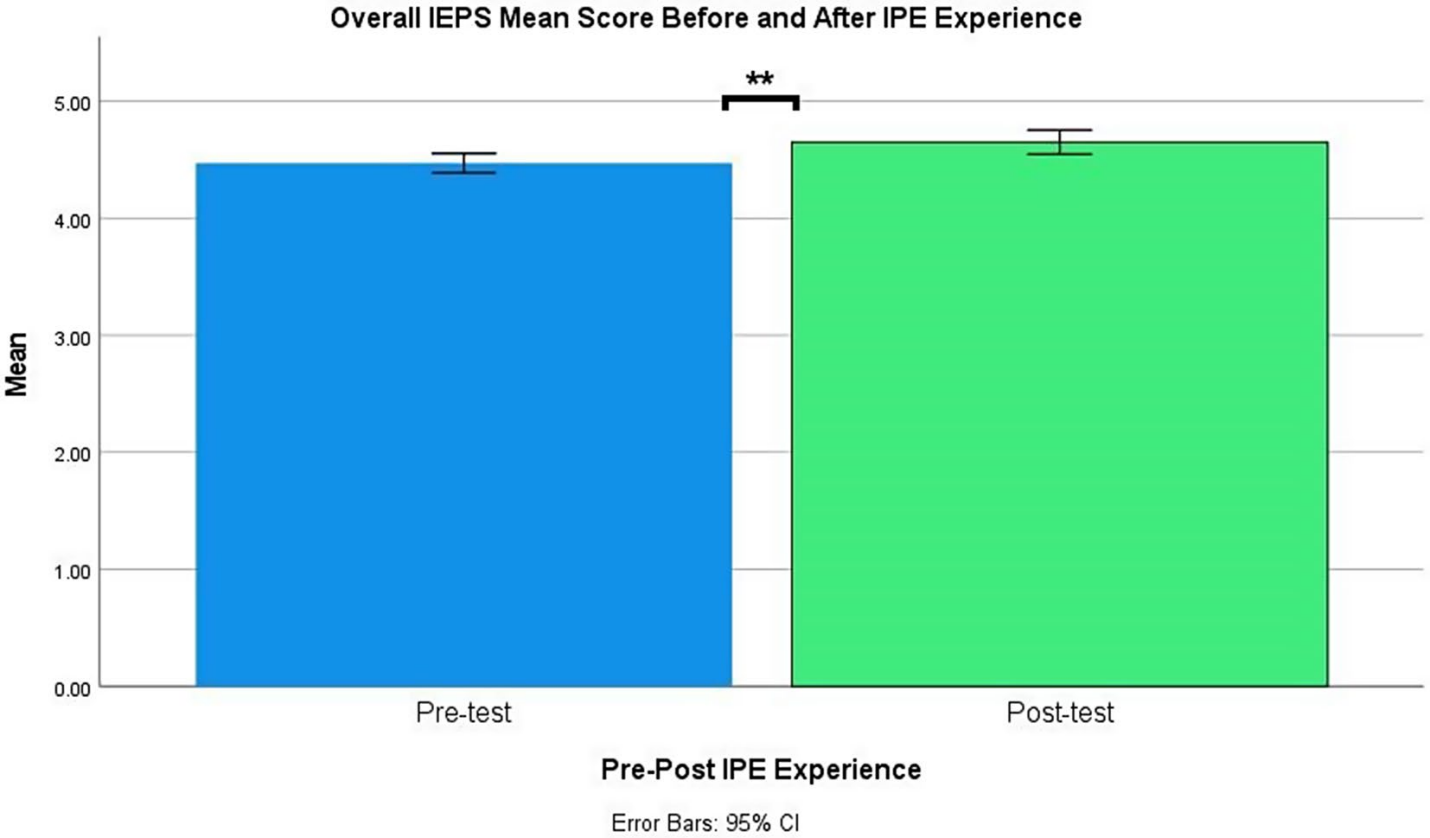
Overall EPIS mean score pre- and post-IPE experience. *Note* Asterisks (**) denote significant differences with *p* < .01. IEPS: Interdisciplinary Education Perception Scale, IPE: Interprofessional Education

**Fig. 3:**
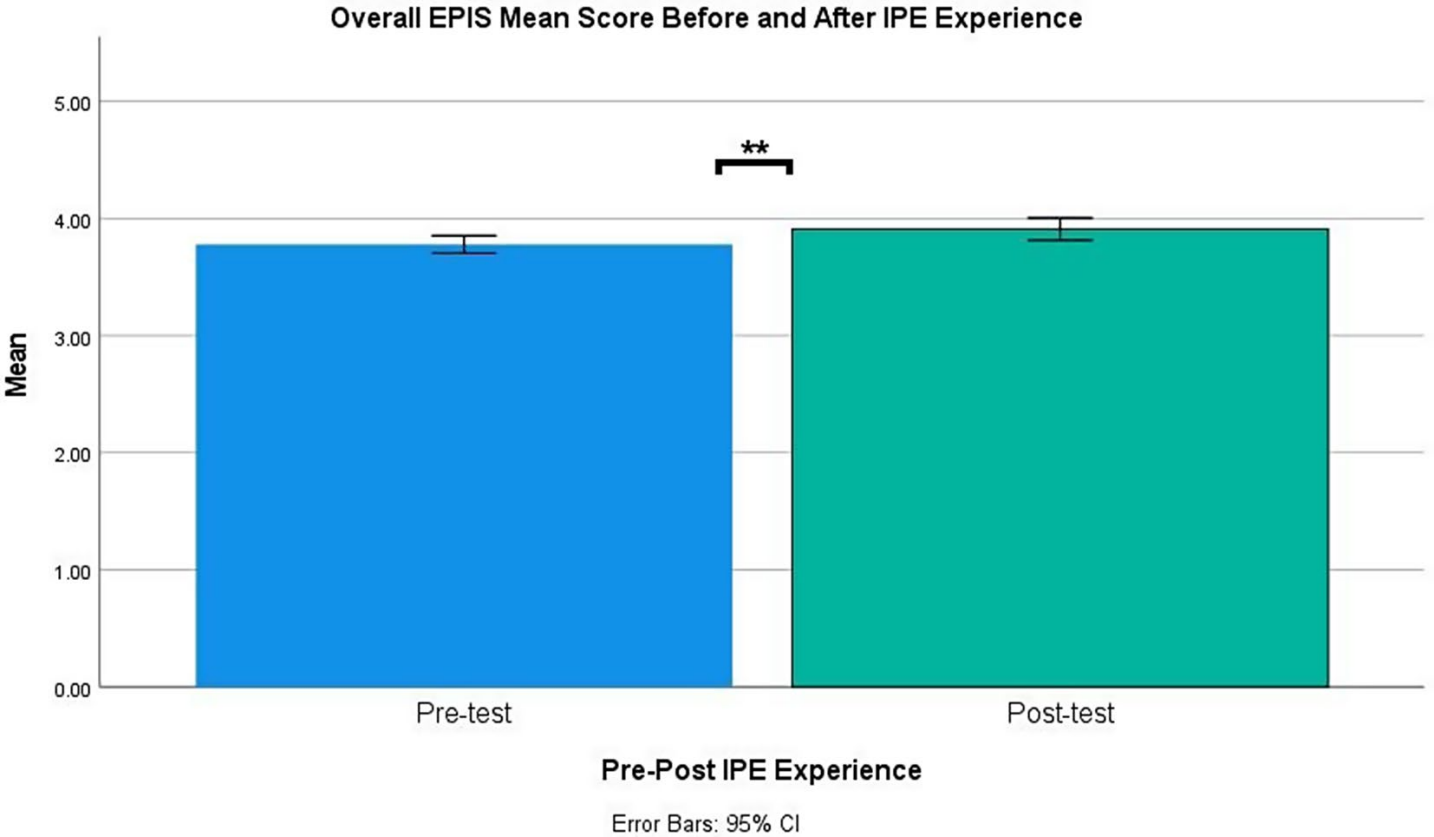
Overall IEPS mean score pre- and post-IPE experience. *Note* Asterisks (**) denote significant differences with *p* < .01. EPIS: Extended Professional Identity Scale; IPE: Interprofessional Education

Table 2; Figs. 2 and 3, show the differences between the collaboration perception and interprofessional identity before and after participating in the IPE PRAE. There was a significant increase between the participants’ pre-test (*M* = 4.47, *SD* = 0.68) and post-test (*M* = 4.65, *SD* = 0.85) mean global collaboration perception scores, *p* < .001; *d* = 0.21; and pre-test (*M* = 3.78, *SD* = 0.61) and post-test (*M* = 3.91, *SD* = 0.77) global interprofessional identity scores, *p* = .006; *d* = 0.17. Overall collaboration perception and interprofessional identity significantly improved after the IPE experience.

**Table 2:** Changes between the interprofessional identity and collaboration perception before and after participating in the IPE program

Variable	Pre-test		Post-test		M_diff_ (SD_diff_)	95% Confidence Interval of the Difference		*t*(262)	*p*	Cohen’s *d*	
	*M*	*SD*	*M*	*SD*		Lower	Upper				
**Collaboration Perception**											
Global score	4.47	0.68	4.65	0.85	0.18 (0.85)	0.12	0.28	3.41	< 0.001	0.21	
**Interprofessional Identity** Global score	3.78	0.61	3.91	0.77	0.13 (0.78)	0.04	0.23	2.26	0.006	0.17	
Interprofessional Belonging	3.72	0.69	3.86	0.81	0.14 (0.86)	0.04	0.25	2.67	0.008	0.17	
Interprofessional Commitment	3.68	0.67	3.87	0.83	0.18 (0.83)	0.08	0.28	3.54	< 0.001	0.22	
Interprofessional Beliefs	3.93	0.66	4.00	0.80	0.07 (0.84)	-0.03	0.17	1.38	0.08	0.09	

*Note* M_diff_ = Mean difference; SD_diff_ = SD difference, IPE: Interprofessional Education

**Fig. 4:**
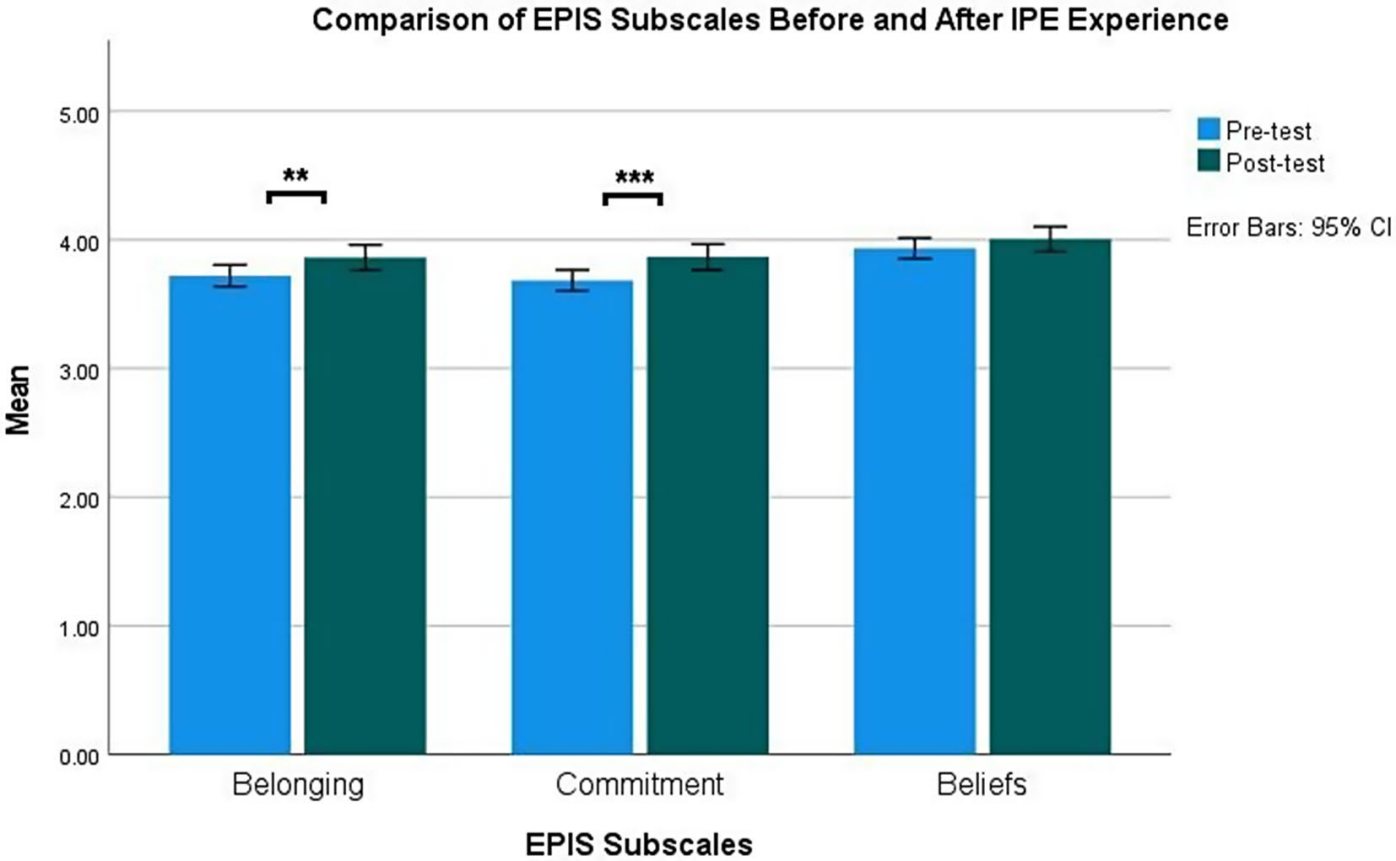
Comparison of EPIS subscales pre- and post-IPE experience. *Note* Subscales with asterisks (**) and (***) denote significant mean differences with *p* < *.01* and *p* < .001, respectively. EPIS: Extended Professional Identity Scale; IPE: Interprofessional Education

Except for the interprofessional beliefs, for which teams’ mean score was already high in T_1_, the mean pretest-posttest differences on interprofessional belonging and commitment were all significant, indicating improvement after IPE participation (Table 2; Fig. 4). Specifically, interprofessional belonging [Pre-test: 3.72 (0.69) vs. Post-test: 3.86 (0.81), *p* = .008; *d* = 0.17] and interprofessional commitment mean scores significantly increased after participating in IPE [Pre-test: 3.68 (0.67) vs. Post-test: 3.87 (0.83), *p* = < 0.001; *d* = 0.22].

### Changes in team-level collaboration perception and interprofessional identity across the IPE program

**Fig. 5:**
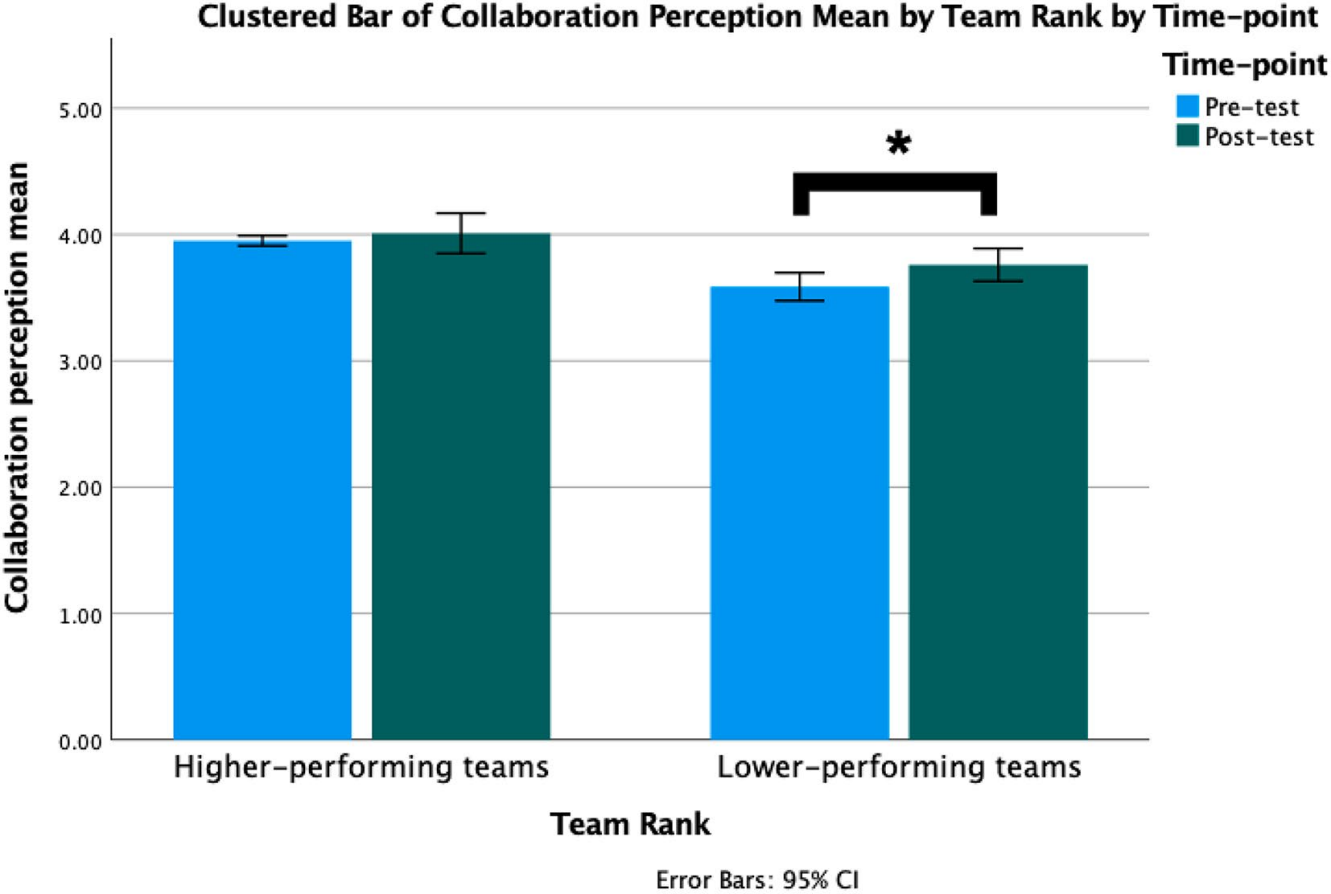
Comparison of higher-performing (i.e., Top 20) vs. lower-performing (i.e., Bottom 20) teams’ collaboration perception before and after participating in the IPE program. *Note* IPE: Interprofessional Education

**Fig. 6:**
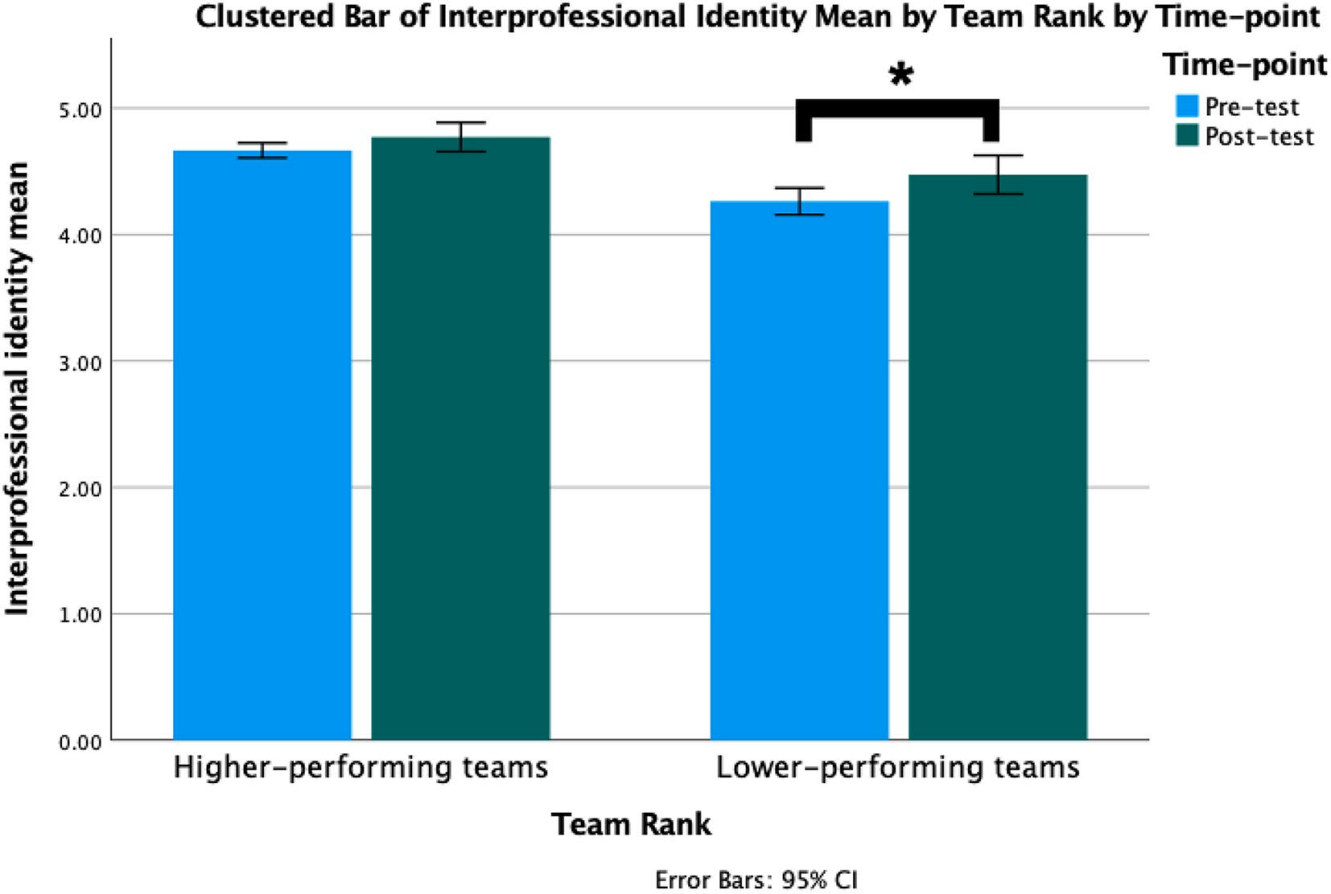
Comparison of higher-performing (i.e., Top 20) vs. lower-performing (i.e., Bottom 20) teams’ interprofessional identity before and after participating in the IPE program. *Note* IPE: Interprofessional Education

Our results indicate that higher-performing (i.e., Top 20) teams did not show significant differences in team-level collaboration perception and interprofessional identity between Time 1 and Time 2. However, lower-performing (i.e., Bottom 20) teams demonstrated a significant difference in team-level mean global interdisciplinary collaboration perception, with a higher collaboration perception at Time 2 (*M* = 3.76, *SD* = 0.28) compared to Time 1 (*M* = 3.59, *SD* = 0.24), *p* = .02; *t* = 2.63; *d* = 0.59 (see Table 3; Fig. 5). Similarly, lower-performing teams also displayed a significant difference in team-level mean global interprofessional identity, showing an increase at Time 2 (*M =* 3.76, *SD* = 0.28) compared to Time 1 (*M* = 3.59, *SD* = 0.24), *p* < .01; *t* = 4.86; *d* = 1.09 (see Table 4; Fig. 6).

**Table 3:** Mean differences in team-level collaboration perception scores across two time points (*n* = 40)

Variable	Pre-test		Post-test		M_diff_ (SD_diff_)	95% Confidence Interval of the Difference		*t* (19)	*p*	Cohen’s *d*
	*M*	*SD*	*M*	*SD*		Lower	Upper			
**Higher-performing teams** (*n* = 20) Team-level collaboration perception	3.95	0.09	4.01	0.34	0.06 (0.34)	-0.10	0.22	0.761	0.23	0.17
**Lower-performing teams** (*n* = 20) Team-level collaboration perception	3.59	0.24	3.76	0.28	0.17 (0.29)	0.04	0.31	2.63	0.02^*^	0.59

*Note* M_diff_ = Mean difference; SD_diff_ = SD difference; Teams were ranked according to pre-test team-level means from 1 (highest) to 40 (lowest). Higher-performing teams are the teams ranked 1–20 (in rank order) include: Teams 11, 37, 34, 6, 20, 5, 2, 22, 4, 27, 8, 14, 9, 7, 25, 36, 40, 26, 17, 33. Lower-performing teams are the teams ranked 21–40 (in rank order) include: Teams 28, 12, 21, 13, 39, 30, 35, 32, 38, 23, 24, 16, 1, 15, 19, 3, 18, 31, 10, 29. A higher team ranking indicates greater interdisciplinary perception. * = *p* < .05

**Table 4:** Mean differences in team-level interprofessional identity scores across two time points (*n* = 40)

Variable	Pre-test		Post-test		M_diff_ (SD_diff_)	95% Confidence Interval of the Difference		*t*(19)	*p*	Cohen’s *d*
	*M*	*SD*	*M*	*SD*		Lower	Upper			
**Higher-performing teams** (*n* = 20) Team-level interprofessional identity	3.95	0.08	3.80	0.39	0.15 (0.39)	-0.03	0.33	1.72	0.051	0.38
**Lower-performing teams** (*n* = 20) Team-level interprofessional identity	3.59	0.24	3.97	0.24	0.38 (0.35)	0.22	0.55	4.86	< 0.001	1.09

*Note* M_diff_ = Mean difference; SD_diff_ = SD difference; Teams were ranked according to pre-test team-level means from 1 (highest) to 40 (lowest). Higher-performing teams are the teams ranked 1–20 (in rank order) include Teams 11, 37, 6, 34, 20, 5, 2, 22, 4, 8, 14, 27, 9, 7, 40, 25, 36, 26, 17, 33. Lower-performing teams are the teams ranked 21–40 (in rank order) include Teams 28, 12, 21, 13, 39, 30, 35, 32, 23, 38, 24, 16, 1, 15, 19, 3, 18, 31, 10, 29. A higher team ranking indicates greater interprofessional identity

### Relationship between collaboration perception and interprofessional identity in pre- and post-IPE

The findings revealed significant and positive correlations between pre-test and post-test global interprofessional identity, its subscales, and interdisciplinary collaboration perception (*p* < .001). The correlation coefficients across all global scales and subscales ranged from *r* = .24 to *r* = .97, indicating moderate to strong positive relationships (see Table 5). The relationship between collaboration perception and interprofessional identity strengthened from the pre-test (*r* = .69; *p* < .001) to the post-test (*r* = .79; *p* < .001).

**Table 5:** Bivariate correlations between pre- and post-test variables and internal reliability coefficients of each global scales and subscales

Variable	*M*	*SD*	1	2	3	4	5	6	7	8	9	10
Pre-test												
1. Collaboration perception T1	4.47	0.68	(0.96)									
2. Interprofessional identity T1	3.78	0.61	0.69^*^	(0.95)								
3. Interprofessional belonging T1	3.72	0.69	0.61^*^	0.92^*^	(0.91)							
4. Interprofessional commitment T1	3.68	0.67	0.65^*^	0.92^*^	0.80^*^	(0.90)						
5. Interprofessional beliefs T1	3.93	0.66	0.61^*^	0.88^*^	0.68^*^	0.70^*^	(0.93)					
***Post-test***												
6. Collaboration perception T2	4.65	0.85	0.40^*^	0.38^*^	0.36^*^	0.39^*^	0.29^*^	(0.97)				
7. Interprofessional identity T2	3.91	0.77	0.34^*^	0.39^*^	0.39^*^	0.38^*^	0.30^*^	0.79^*^	(0.98)			
8. Interprofessional belonging T2	3.86	0.81	0.33^*^	0.36^*^	0.35^*^	0.36^*^	0.28^*^	0.76^*^	0.97^*^	(0.94)		
9. Interprofessional commitment T2	3.87	0.83	0.34^*^	0.37^*^	0.38^*^	0.40^*^	0.24^*^	0.77^*^	0.96^*^	0.91^*^	(0.96)	
10. Interprofessional beliefs T2	4.00	0.80	0.31^*^	0.39^*^	0.38^*^	0.32^*^	0.35^*^	0.74^*^	0.94^*^	0.85^*^	0.83^*^	(0.95)

*Note* T1 = Time 1; T2 = Time 2. Scores shown in parentheses on the diagonal are internal consistency reliabilities of the scales (Cronbach’s alpha). *p < .001 level

Interdisciplinary collaboration perception scores in Time 1 significantly predicted the variance in interprofessional identity scores in Time 2 (β = 0.347 [95% C.I. = 0.265–0.521], *p* < .001), controlling for age, gender, and year level of the participants. The regression results indicated that interprofessional perception in Time 1 and the controlled demographic variables explained 14.5% of the variance in interprofessional identity scores in Time 2 (*R*^2^ = 0.145, *F*(4,258) = 10.941, *p* < .001).

Additionally, team-level correlational analysis revealed a significant positive correlation between the collaboration perception and interprofessional identity of lower-performing teams in Time 1 (*r* = .93, *p* < .001). Similarly, higher-performing teams exhibited a significant positive correlation between collaboration perception and interprofessional identity in Time 1 (*r* = .94, *p* < .001). However, no significant correlation results were observed in Time 2.

### Qualitative results

#### Student IPE outcome expectations

There were 263 students who responded to the open-ended questions. Prior to participate the IPE program, students were asked to comment on their expectations for the program. Within the categories, there are three themes with 11 sub-themes (Appendix 1). Students’ written reflections indicated that they expected the IPE program could help form their interprofessional identity (56%), promote interprofessional perceptions and competencies (23.8%), and acquire knowledge, skills, and experiences (20.2%). Regarding the code frequency that falls within each theme, students expected the IPE PRAE to promote interprofessional commitment the most (33.1%) and improve their communication skills (20.8%). Around 20.8% of the students believed that the IPE PRAE improved their communication skills. Approximately 42.6% of students stated that it was their first time participating in a program that required them to work with other professions. One student wrote: *“The most important thing I hope to learn is how to collaborate…*,* I hope that I can build connections*,* develop interpersonal skills*,* as well as gain knowledge in relation to the health care sector. I look forward to working with my diverse team members!”* (Law, Year 3).

### Perceived program outcomes

After the program, the majority of students (85.2%) perceived the whole IPE learning process as meeting their expectations. One student stated, “*I think the whole learning process met my expectations to a large extent. I learnt a lot of medical knowledge. I was impressed by my teammate’s professionalism.*.*”* (Nursing, Year 3). However, it is worth noting that some students raised concerns and provided constructive feedback in response to open-ended questions. Common concerns included teammates being too reserved to communicate effectively and a tendency to focus solely on their own professions, which hindered collaborative efforts. Students offered suggestions for improvement, such as incorporating more hands-on activities, real-life simulations, and strategies for conflict resolution. Additionally, logistical issues were mentioned, and some students proposed the utilization of online meetings as a potential solution.

### Beneficial dimensions of the IPE program

The beneficial dimensions of IPE PRAE as perceived by participants were subsumed under five major themes. The majority of students stated that the program facilitated the development of interprofessional identity through the development of interprofessional belonging (49.1%), commitment (26.8%), and belief (2.7%). There were 36.4% of students who stressed that they could learn from and collaborate with students from other health professions. Around 12.7% of students expressed that IPE is a vehicle to meet people from other health professions and make new friends. Students reported being provided a range of opportunities to collaborate in a formal and informal manner. In describing the importance of IPE experience as interprofessional socialisation shaping their interprofessional identity, one student stated that: *“I learned to view patient care not only from the medical side of things but also from social and legal perspectives. This experience was highly inspiring and educational; it taught me how to collaborate with my team members…I am now better equipped to work with other interprofessional teams in the future.”*(Social work, Year 3).

In terms of interprofessional commitment, students perceived that they could identify themselves as a part of the interprofessional team and that they preferred to work with others. One student stated that *“It was the first time that I worked with individuals of other professions*,* which provided me insight into how it actually works.”* (MBBS, Year 5). Moreover, through IPE, students also recognized that their interprofessional beliefs have been reinforced: they can understand how to set common goals, make joint decisions, and strive for consensus when they work together to manage patients. One student reported that “*Different professions have their own focus*,* and only these varied opinions can help develop a more inclusive care plan. Patients’ benefits are maximized.”*(Chinese Medicine, Year 5). Additionally, 16.3% of students reported that the program improved their interprofessional competencies and perceptions. Students highlighted that they could develop communication skills, understand how to construct the care plan, and strengthen their awareness of interprofessional collaboration for patient treatment. Furthermore, 5.1% of students recognized the IPE learning experience as valuable and enjoyable. They acquired real workplace experience, managed time effectively, and learned content knowledge. One student mentioned that *“I was able to directly communicate with professionals of different disciplines*,* which was a very precious learning opportunity.”* (Speech and Hearing Sciences, Year 3).

## Discussion

**Fig. 7:**
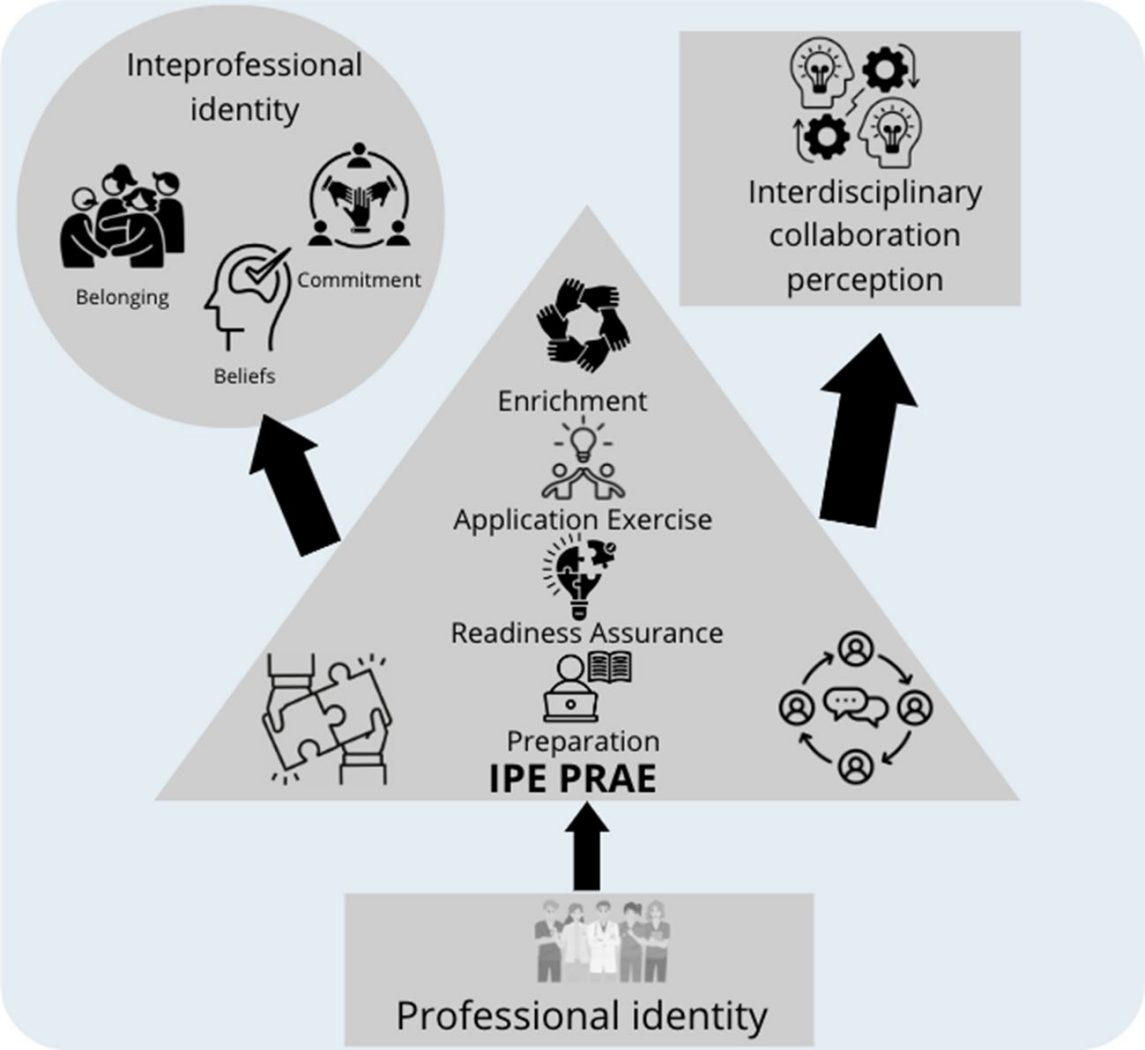
How the IPE serves as the contextual trigger for shaping students’ interprofessional identity and collaboration perception. *Note* IPE: Interprofessional Education

This study presents an empirical investigation that explores the impact of an IPE program on the transformation of health profession students’ collaboration perception and interprofessional identity. The results, both from quantitative and qualitative analyses, provide promising evidence that the IPE program serves as a contextual trigger, effectively fostering students’ interprofessional collaboration perception and interprofessional identity (Fig. 7). Following the program, students acknowledged the value of the entire learning process in facilitating cross-disciplinary learning and influencing their interprofessional identity. Notably, teams with initially low collaboration perception and interprofessional identity demonstrated a significant increase after their participation in the IPE program.

### Beyond labels: interprofessional identity formation and collaboration attitudes improvement

Encouragingly, we found that after participating the IPE program, a significant improvement in students’ interprofessional collaboration perception and interprofessional identity from Time 1 to Time 2 can be detected. Particularly, students’ interprofessional commitment and belonging showed positive development. Through active engagement in collaborative teamwork, communication, and joint formulation of health management plans within the IPE program, students experienced enhanced social inclusiveness within their own profession, a strengthened sense of belonging in the interprofessional team, and improved collaboration with other professions. Furthermore, at the team level, the lower-performing teams exhibited a significant improvement in interprofessional collaboration perception and interprofessional identity compared to the higher-performing teams. The qualitative findings provide additional support for the quantitative results. Notably, students from lower-performing teams articulated a more comprehensive and specific appreciation for the benefits of IPE. Their insights included the recognition of the advantages of integrating diverse ideas to formulate patient care management plans, the value of interacting with peers from different professions that they typically do not encounter, and a heightened emphasis on the importance of ‘team collaboration’ and ‘working with others’. Upon recognizing the benefits of interprofessional collaboration, they may have been more receptive to new ideas and approaches, thereby fostering a more cohesive and dynamic learning environment. Moreover, these students demonstrated a deep-rooted commitment to effectively learn, communication, teamwork, and collaboration within interprofessional teams. Their interactions with peers from varied professions likely sparked novel insights and facilitated a richer exchange of knowledge and experiences, leading to enhanced collaboration attitudes and a stronger sense of interprofessional identity. This proactive engagement with the core tenets of interprofessional education could have fuelled their growth and development throughout the program, resulting in more pronounced improvements in their collaboration attitudes and interprofessional identity.

In contrast to earlier findings that indicated non-significant [27] or declining tendencies in interprofessional identity over time [7], our findings indicate otherwise. This difference could be attributed to the developmental stage of the students. Junior-year health profession students are typically in the stage of independent operation and may lack awareness and understanding of the importance of team collaboration, which can influence their interprofessional identity [7]. However, the senior-year students in our sample demonstrated a conscious effort to develop their interprofessional identity through IPE. Also, while previous studies have highlighted that an IPE intervention can influence professional identity formation [13, 28], our quantitative and qualitative findings add new insights that IPE can also influence, or more specifically enhance, interprofessional identity and interprofessional collaboration perception. As such, understanding the effect of participating in IPE on health profession students’ training at various stages of learning and their interprofessional identity could shed light on how educators can effectively incorporate an interprofessional curriculum into profession-specific curricula [7]. Doing so would enable students to develop both professional and interprofessional identities upon graduation.

Furthermore, our findings support previous studies in the field, such as in one study [29] which demonstrated the positive impact of interprofessional learning on the attitudes and perceptions towards collaboration of health profession students. However, it is worthwhile to note that some studies have reported a decline in students’ attitudes towards interprofessional learning [30], while others have found no significant changes after the interprofessional learning (e.g., Lockeman et al. [31]). In our study, the immersive and intensive interprofessional learning experience, involving a diverse group of health professionals collaborating to manage a patient case, fostered a distinct understanding of interprofessional collaboration. This finding strongly supports the notion that IPE serves as a motivational context, prompting students to recognize the significance of collaboration with professionals from other disciplines [32].

Our qualitative findings corroborate the aforementioned quantitative results where students’ responses formed themes that corresponded to the three dimensions of Reinders [14], interprofessional identity model. Specifically, the majority of students reported that the IPE learning experience facilitated interprofessional belonging, in which they met, learned and collaborated with people from other health professions. Additionally, students also identified themselves as part of an IPE team and preferred to work with others in an interprofessional team, indicating the enhancement of interprofessional commitment. Through the program, students gradually formed interprofessional reliance, which began to shape their attitudes and perceptions and resulted in an appreciation of interprofessional collaboration.

### Building bridge: establish the connection between collaboration perception and interprofessional identity

Social identity theory provides a clue that an individual’s perception of their future roles contributes to the promotion of their identity [33]. We based this assumption on the idea that “maintenance of identities are guided partly through perceptions of oneself, other people, and situations” [18](p21). In support of social identity theory assertions, our results suggest that students’ collaboration perception plays a crucial role in the development of their interprofessional identity, and the positive correlation would be strengthened through interprofessional learning. Qualitative findings also supported the quantitative results, manifesting that the majority of students noted that the IPE program provides a learning platform where they can collaborate, communicate with, and acquire new insights and knowledge from different professions. Such learning experiences also strengthen awareness of the importance of interprofessional collaboration for patient treatment. The more they have actual collaborations with other professions, the more interprofessional belonging and commitment they receive from the IPE team. In turn, upon the formation of the interprofessional identity, students are prone to have a positive collaboration perception towards IPE, which helps develop interdisciplinary autonomy and competence as well as actual cooperation.

### Breaking barriers: IPE serving as the contextual trigger

Identity development is a complex process influenced by contextual triggers, as beliefs alone may not translate into actions unless integrated into one’s identity. Previous research has highlighted the importance of regular and high-quality interprofessional contact in preventing a decline in interprofessional identity [7]. The qualitative analysis of students’ responses to the open-ended questions regarding the beneficial dimensions of the IPE program revealed a positive perception of incorporating sequential collaboration-oriented activities as a means to facilitate the development of interprofessional identity and improve collaboration attitudes. In line with the IPE implementation strategies proposed by Diggele et al. [34], we utilized an online learning forum for pre-class material discussions, fostering team cohesiveness through the collaborative formulation of team names. Small-group meetings allowed students to collectively analyze case scenarios, develop patient management plans, complete group assignments, and engage in team learning reflection. Encouraging within-group and between-group interactions proved effective in enhancing students’ interprofessional identity and interdisciplinary perceptions. To promote such interactions, it is crucial to provide formal and informal opportunities for interprofessional engagement [34]. Notably, Labrague et al. [35] emphasized the benefits of simulation in fostering interprofessional communication, recognition of professional responsibilities, teamwork, and professional self-assurance. We therefore designed our IPE program to revolve around simulation-based learning activities. This approach directly focused on clarifying roles and responsibilities within a given setting, dispelling negative stereotypes, and providing students with valuable practice before entering real work placements. Qualitative analysis revealed that students valued this collaborative learning experience and displayed a genuine interest in understanding other professions.

The implications of this study are significant for educators, curriculum designers, and policymakers in the field of health professions education. Incorporating an IPE program into the curriculum can effectively enhance students’ collaboration perception and interprofessional identity, ultimately preparing them for collaborative practice in the healthcare system. By engaging students in interprofessional teamwork, communication, and joint decision-making processes, the IPE program provide a valuable context for students to develop a sense of belonging and commitment to interprofessional collaboration. These programs can help break down professional silos and promote a collaborative culture among future healthcare professionals. Educators should consider integrating IPE experiences throughout the curriculum, starting from the early stages of professional education, to ensure that students have ample opportunities to develop their interdisciplinary perception and interprofessional identity over time.

However, it is important to acknowledge the limitations of this study. Firstly, the study relied on self-report measures, which are subject to social desirability bias. Future research could incorporate objective measures or observational data to complement self-report data. Additionally, the study involved only one cohort of IPE students without a control group, although data were collected at two time points (pre-test and post-test) using a mixed-method design. Future studies could employ a longitudinal study design to explore the long-term effects of IPE on promoting interprofessional identity and collaboration perceptions. Follow-up studies that assess the sustainability of these effects beyond the immediate post-intervention period would be valuable.

## Conclusion

This study shows encouraging results that have impacts theoretically, methodologically, and practically. *Theoretically*, we stimulated a discussion on the conceptual link between collaboration perception and interprofessional identity where our empirical data established their relations at both individual and team levels. *Methodologically*, we used a concurrent triangulation mixed-method design which allowed our research questions to be studied from different perspectives complementing the strengths of each perspective [36]. *Practically*, this study contributes to the understanding of the effects of IPE on the formation of students’ interprofessional identity and collaboration perception through deliberate IPE program design. Both quantitative and qualitative data lend support to our understanding that interprofessional identity is a malleable construct that can be improved. For the IPE community of practice, we hope that our effort to understand how to best design IPE to develop desirable outcomes (e.g., interprofessional identity) will get their needed attention to enable us to elevate further the discussion of IPE as a contextual trigger for facilitating students’ collaboration perception and interprofessional identity formation.

### Electronic supplementary material

Below is the link to the electronic supplementary material.

Supplementary Material 1

## Data Availability

The data that support the findings of this study can be requested from the corresponding author upon reasonable request.

## Abbreviations

IPE: Interprofessional Education
EPIT: Extended Professional Identity Theory
PRAE: Preparation, Readiness Assurance, Application Exercise, Enrichment Activity
MBBS: Bachelor of Medicine, Bachelor of Surgery
IEPS: Interdisciplinary Education Perception Scale
EPIS: Extended Professional Identity Scale
RAT MCQs: Readiness Assurance Test Multiple Choice Questions
AE: Application Exercise
