# Supplementary material for: Unveiling the impact of interprofessional education on shaping students’ interprofessional identity and collaboration perception: a mixed-method study
Source: BMC Med Educ. 2024 Aug 8;24:855. doi: 10.1186/s12909-024-05833-0 (PMC11312693; doi:10.1186/s12909-024-05833-0)
Supplement: Supplementary file 1 — Supplementary Material 1 [file 12909_2024_5833_MOESM1_ESM.docx]

Appendix 1. Summary of the categories, themes, sub-themes, code frequency, and example of illustrative quotes for the qualitative data

| **Categories** | **Theme** | **Sub-theme** | **Code Frequency** | **Example of illustrative quotes** |
| --- | --- | --- | --- | --- |
| Outcome  Expectations towards the IPE programme | Promote  interprofessional perceptions and competencies | Improve communication  skills | 71 | “*Communication skills are very important when it comes to taking care of patients, so I hope I can*  *improve more on this*  *aspect*.” |
|  |  | Establish mutual respect | 6 | “*To respect every profession and listen to their advice humbl*y” |
|  |  | Develop interpersonal and intrapersonal skills | 4 | “*I hope to learn to enhance both the intrapersonal and interpersonal skills during these collaborations*.” |
|  | Improve interprofessional identity | Interprofessional commitment | 113 | “*I want to work with people from different health professions and I believe that it may help me to cooperate with other interprofessional teams in my future career path*.” |
|  |  | Interprofessional belonging | 59 | “*Making new friends from different professionals. Understanding their roles in the healthcare setting*.” |
|  |  | Interprofessional belief | 19 | *“Consensus-building E.g. There may be slight conflicts between different theoretical schools/practices/common approaches/professional values under different professions”* |
|  | Acquire knowledge, skills, and experiences | Acquire knowledge and experience on how the health profession works | 30 | *“Basic knowledge about medical negligence and how the health profession works in Hong Kong.”* |
|  |  | Learn how to provide optimal care to patients | 21 | *“How to treat a patient in a more holistic view and apply the ICF framework to optimize patients' quality of life.”* |
|  |  | Formulate the care plan | 11 | *“How to communicate and collaborate with other professions to devise a*  *good care plan for the patient.”* |
|  |  | Develop interpersonal skills to work with | 4 | *“Through IPE, we can learn many soft skills and* |
|  |  | individuals in different professions |  | *prepare ourselves for the working environment in the future.”* |
|  |  | Manage time | 3 | *“Time management; many things to do already”* |
|  | Subtotal |  | 341 |  |
| Beneficial aspect of the IPE programme | Interprofessional belonging | Learning and collaborating with people from other health professions | 121 | *“Team collaboration, gaining knowledge from others”* |
|  |  | Meeting and getting to know people from other health professions | 42 | *“IPE program provides a chance for me to meet nice, responsible people from other departments.”* |
|  | Interprofessional commitment | Identity oneself is part of an IPE team | 13 | *“Got to know more about how to work in a clinical team as a social worker.”* |
|  |  | Working with others in an interprofessional team | 76 | *“In the future, I will use what I have learnt in IPE to collaborate with colleagues, to provide a holistic approach to patients as a multidisciplinary team”* |
|  | Interprofessional belief | Setting common goals | 3 | *“Setting common care goals through interprofessional cooperation.”* |
|  |  | Jointly decision making | 3 | *“I have understood that a comprehensive healthcare plan for patients must be jointly discussed.”* |
|  |  | Strive for consensus | 3 | *“Yes, the IPE platform did provide a platform for people from many healthcare-related disciplines to work together, from receiving the case of Aston, analysis, and starting to form a management plan. Meanwhile, discussion and consensus are needed.”* |
|  | Improve interprofessional competencies and perceptions | Develop communication skill | 26 | *“Having actual experience of communicating with other health care providers”* |
|  |  | Understand how to construct the care plan for patients | 18 | *“Getting to develop a management plan together*  *like what people do in real*  *life.”* |
|  |  | Strengthen the awareness of interprofessional collaboration for patient treatment | 10 | *“The patients in the current healthcare system can benefit from the IPE most. When students perform collaborative work, patients can have comprehensive care. Besides the medical treatment from doctors and nurses, their psychological needs are also valued. The patients can receive holistic care by implementing services from different clinical disciplines through collaborative work. They can benefit the most.”* |
|  | Subtotal |  | 332 |  |
